# Presentations and management of different causes of chylothorax in children: one medical center’s experience

**DOI:** 10.1051/bmdcn/2017070105

**Published:** 2017-03-03

**Authors:** Chien-Heng Lin, Wei-Ching Lin, Jeng-Sheng Chang

**Affiliations:** 1 Division of Pediatric Pulmonology, China Medical University Children’s Hospital Taichung 404 Taiwan; 2 Department of Biomedical Imaging and Radiological Science, College of Health Care to College of Medicine, China Medical University Taichung 404 Taiwan; 3 Department of Radiology, China Medical University Hospital Taichung 404 Taiwan; 4 School of Medicine, College of Medicine, China Medical University Taichung 404 Taiwan; 5 Division of Pediatric Cardiology, China Medical University Children’s Hospital Taichung 404 Taiwan

**Keywords:** Chylothorax, Management, Etiology, Children

## Abstract

Background: Chylothorax in children is a relatively rare cause of pleural effusion. However, it is usually a common complication of cardiothoracic operations like open-heart surgery. Other etiologies for chylothorax, such as trauma or malignancy, occur more common in adults and rare in children. To explore the etiologies of chylothorax in children, this study analyzed the pediatric patients that were admitted in to onea medical center.

Methods: We retrospectively reviewed the medical records of the pediatric patients that were admitted to this tertiary transfer center with a diagnosis of chylothorax during the period of 1995 to 2005.

Results: A total of 22 patients (15 females and 7 males) with chylothorax were enrolled in our study. The etiologies for chylothorax were the following: a complication of cardiothoracic surgery in 14 patients (63.6%), congenital chylothorax in 5 patients (22.7%), association with neuroblastoma in 2 patients (9.1%), and congenital nephrotic syndrome in 1 patient (4.6%). All patients required medical therapy. Chest tube drainage was necessary to provide for twenty patients (90.9%), and surgical intervention was necessary to perform for 3 patients (13.6%). Four patients (18.2%) expired due to other causes.

Conclusion: Cardiothoracic surgery was the most common cause of chylothorax in children at the institution surveyed. Medication and chest tube drainage were effective in treating most of these chylothorax-afflicted patients. In addition, early recognition, medication, and performing surgical intervention when necessary are important measures to avoid a catastrophe.

## 1. Introduction

Chylothorax is a condition of thoracic duct damage and chyle leakage from the lymphatic system into the pleural space. A diagnosis of chylothorax is made when an analysis of the pleural fluid content finds that triglyceride > 1.1 mmol/*l*, the absolute white cell count > 1,000/ mm^3^, with a lymphocyte fraction > 80% [1, 2].

The etiologies of chylothorax in children are usually divided into four categories: trauma (including surgery), idiopathic or congenital, malignancy, and miscellaneous disorders [3]. The clinical courses vary substantially among the different causes of chylotho rax. Postoperative complications of cardiothoracic surgery are the leading causes of chylothorax in children [2]. The congenital malformations of the pulmonary or thoracic lymphatic system, which are associated with some dysmorphic syndromes, are relatively rare causes for chylothorax [2, 4]. In adults, thoracic or neck trauma, or tumors occupying the upper thoracic aperture are the most common causes of chylothorax [5–7]. Treatment of chylothorax includes repairing the underlying condition(s), giving proper medication, and surgical intervention(s) [2, 4].

This study is aimed at reviewing pertinent medical records to explore the etiologies and characteristics of patients of chylothorax that were admitted to a medical center.

## 2. Materials and methods

We retrospectively collected and analyzed all of the patients with a chylothorax diagnosis in our hospital database from the years 1995 to 2005. Clinical information of age, sex, gestation age, underlying disease(s), clinical presentation, laboratory data of pleural fluid, duration of chest tube drainage, volume of pleuraleffusion, associated disease(s) and their management, any complications, and mortality were obtained from the review of relevant charts.

Medical therapy consisted of nothing per mouth (NPO) at the beginning of treatment and then fed with formula of a medium chain triglycerides oil (MCT oil). Agents such as somatostatin and octreotide were used with all patients. When MCT oil formula failed to stop the occurrence of chylothorax, the patients were again treated with NPO and supported by total parenteral nutrition (TPN). The decision to set up chest tubes for drainage of pleural fluid was made by clinical physicians in accordance with the status of each patient’s respiratory distress.

## 3. Results

The median age of the 22 total patients was 11.5 months, with their range being from 1 day to 3 years. The female to male ratio was 15 to 7. Seven patients suffered right side chylothorax (32%), nine suffered left side (41%), and 6 had bilateral (27%). Aside from two patients with diagnostic tapping, the first day drainage of chyle in the other 20 patients was between 33 to 890 *ml* (median: 240 *ml*).

All patients were treated initially with NPO, a diet of low fat medium chain triglycerides, and TPN. The median and mean duration of chest tube drainage in the 20 patients was 20 and 21.2 days, respectively (with a range of 2 to 31 days). The mean drainage duration in in cardiothoracic surgery group, congenital chylothorax group, and neuroblastoma group were 21.2, 20.8, and 25.7 days, respectively. However, there were 3 patients (13.6%) that needed surgical intervention (2 with pleural peritoneal shunt and 1 with pleurodesis) after chest tube drainage was unsuccessful.

Although chylothorax occurred in 14 of the total 22 patients (63.6%) due to a complication from cardiothoracic surgery (Table 1), it occurred in only 3.8% of all 363 cardiothoracic surgeries that were performed in the time period being considered. The median number of postoperative days before diagnosing clinically evident chylothorax were 4.5 days, though it was noted in 7 patients within the first 48 hours.

**Table 1:** Data of Postoperative chylothorax after cardiothoracic surgery in children.

Case	Age/Sex	Underlying disease	Type of operation	Triglyceride levels in pleural fluid (mmol/l)	Total number of cells and lymphocytes (cells/ml)	Site	Drainage days
1*	5mo/F	VSD + PS, TGA	Total repair	1.8	2350	Left	30
2	1d/F	TGA	Arterial switch	4.4	2550	Right	25
3	1yr/M	VSD	VSD repair	10.5	2398	Bilateral	6
4*	1mo/M	VSD + ASD	Closure of ASD/VSD	2.1	1106	Bilateral	35
5	7mo/F	ECD,	ECD repair	2.5	2650	Right	No drainage
6	1mo/M	CDH	CDH repair	1.4	987	Left	9
7	8mo/F	TOF	TOF repair	5.9	1108	Bilateral	R:30, L:29
8#	4d/F	TAPVR	Correction of TAPVR	14.7	8700	Bilateral	R:32, L:4
9	2yr/F	Large PDA	PDA ligation	1.6	1658	Left	28
10	20d/F	PDA	PDA ligation	8.8	6790	Left	28
11	1d/F	TGA	Arterial switch	1.4	3709	Left	20
12	2yr/M	RV hypoplasia	Hemi-Fontan	2.3	1146	Right	16
13	1yr/F	ECD + TOF	Repair of ECD, TOF	2.2	3799	Left	19
14	3yr/F	VSD	VSD repair	2.3	9780	Left	7

* Performed pleural-peritoneal shunting,

# expired on postoperative day 49

M = male, F = female, R = right, L = left, VSD = ventricular septal defect, ASD = atrial septal defect, EDC = endocardial cushion defect, CDH = congenital diagram hernia, TOF = Totralogy of Fallot, TAPVR = Total anomalous pulmonary venous return, PDA = patent ductus arteriosus, TGA = transposition of the great arteries, RV = right ventricular.

Congenital chylothorax occurred in five patients (22.7%, Table 2). Two cases were due to a complication from neuroblastoma (9.1%, Table 3), and one (4.6%) was from congenital nephrotic syndrome. Prenatal ultrasound studies observed chylothorax in the other two patients, and sono-guided thoracentesis was undertaken in one of the two fetuses at a gestational age of 32 weeks.

**Table 2:** Data of children with congenital chylothorax.

Case	Age/Sex	GA/BBW (gm)	Triglyceride levels in pleural fluid (mmol/l)	Total number of cells and lymphocytes (cells/ml)	Site	Drainage days
1	4d/M	39/4000	9.8	1460	Left	22
2*	5d/F	39/3250	1.6	2479	Right	No drainage
3	1d/M	38/3305	2.1	3790	Right	25
4	9d/M	38/3200	3.9	5690	Right	5
5	1d/M	37/3350	1.5	1180	Bilateral	31

* Intrapartum sono-guided thoracentesis was performed at GA 32 wks.

**Table 3:** Two children with chylothorax associated with neuroblastomas.

Case	Age/Sex	Triglyceride levels in pleural fluid (mmol/l)	Total number of cells and lymphocytes (cells/ml)	Site	Drainage days	Prognosis
1	2yr/F	16.7	3050	Bilateral	R:30 L:22	Expired on day 55
2	2yr/F	2.9	1580	Left	25	Expired on day 34

The patient with congenital nephrotic syndrome was found to suffer from bilateral chylothorax at birth (male, 3000 g at 36 weeks of gestation). The triglyceride concentration in his pleural fluid was 2.1 mmol/*l*, and the total number of cells in chylous was 4980 cells/*ml*. Because of acute renal failure, a continuous arteriovenous hemofiltration (CAVH) was established soon after birth. However, the pleural effusion continued in large amount, and a pediatric surgeon was consulted for a pleurodesis procedure on day 18 of life. In the end, the patient died of end stage renal failure and refractory hypotension on day 24 of life.

After a 5-year follow-up, eighteen patients are still alive without recurrent chylothorax, and the mortality rate is 18.2% (2 passing away from neuroblastomas, 1 from nephrotic syndrome, and 1 from total anomalous pulmonary venous return). Thus, there was no mortality directly related to the chylothorax.

## 4. Discussion

Although chylothorax is a rare cause of pleural effusion in most children, it is the most common form of pleural effusion in non-surgical neonates [8]. Haines C *et al.* reported incidence of children with chylothorax in the UK was 0.0014% [9]. There are multiple etiologies of chylothorax in children. In this survey, postoperative chylothorax was found to be the most common etiology, followed by congenital chylothorax, malignancy, and miscellaneous disorders.

### 4.1. Postoperative chylothorax

In children, the reported incidence of chylothorax after cardio thoracic surgery is between 0.85% and 6.6% [2, 8]. In a national, multicenter study, which involved 172 children with chylothorax and was reported by Haines C *et al*, the incidence of chylothorax following a cardiac surgical procedure was 3.2%, which is almost the same percentage as our study (3.8%) [9]. In their report, 65.1% of the cases of chylothorax in children were associated with cardiac surgical procedures, with a neonatal diagnosis being the second most common (22.7%) [8]. In our study, the most common etiology was found to be due to the complications of cardiothoracic surgery (63.6%), which is also similar to Haines C *et al.* survey.

A high incidence of chylothorax was observed in heart transplantation and Fontan procedures in a study by Chan EH *et al.* [10] Undoubtedly, heart transplantation is associated with increased trauma to the chest cavity and Fontan (or cavopulmonary anastomosis) procedures will elevate superior vena cava pressure, both of which can result in a higher risk for chylothorax. In the Haines C *et al.* study, the Fontan procedure and repair of Tetralogy of Falot (TOF) are the two most common procedures found to have led to postoperative chylothorax [9]. However, in our study, patent ductus arteriosus ligation and repair of the ventricular septal defect (VSD) are the two most common procedures found to cause postoperative chylothorax. The risk factors for cardiothoracic surgery related chylothorax in children are: the complicated nature of the procedure, secondary chest tube closure, younger age, lower body weight, genetic syndromes, vein thrombosis, lengthy cardiopulmonary bypass, X-clamp time, and higher annual hospital volume [11, 12].

### 4.2. Congenital/idiopathic

Congenital chylothorax can be classified either with congenital lymphatic malformations, such as lymphangiomatosis or lymphangiectasia, or associated with syndromes, such as Down syndrome, Noonan syndrome, Turner syndrome, hydrops fetalis, yellow nail syndrome, and other rare syndromes [4]. In the Haines C *et al.* study, 16.8% of the chylothorax cases had a recognized congenital anomaly, with Down syndrome (10.5%) and Noonan syndrome (4%) [9]. Approximately 5 to 10% of chylothoraces are idiopathic, and the cause(s) in such a setting are unknown [13]. Idiopathic chylothorax is the most common form of pleural effusion in the first few days of life and 50% of newborns with idiopathic chylothorax develop symptoms within 24 hours of birth [13]. As well, cases without a clear explanation for the occurrence of chylothorax can be considered congenital chylothorax. In our study, 5 patients (22.7%) with congenital chylothorax were idiopathic without an associated syndrome.

### 4.3. Malignancy

Malignancies, while one of the most common causes of chylothorax in adults, are a less prevalent cause in children. In the Staats *et al.* series, lymphoma accounted for most cases (52.6%) of chylothorax in adults [15]. In children, lymphoma is also the most common tumor associated with chylothorax (60% to 70% of cases), and it may be the presenting symptom [2, 16, 17]. Other malignancies include neurogenic, teratoma, Wilm, ovarian, and Kaposi sarcoma [2, 4]. In this study, the 2 cases of malignancy-related chylothorax were patients with neuroblastoma, and they both expired.

### 4.4. Miscellaneous disorders

The various other medical conditions that have been associated with chylothorax are classified as miscellaneous, and these miscellaneous causes are more common in adults than children [18]. Granulomatous infections such as tuberculosis, histoplasmosis, and sarcoidosis are associated with chylothorax because of lym padenopathy obstructing the thoracic duct [4, 19, 20]. In some patients with chylous ascites, which in turn is related to a primary abdominal process such as nephrotic syndrome, hypothyroidism, cirrhosis of the liver, abdominal operations, and pancreatitis, chylothorax can occur [18, 21]. Other causes include mediastinal radiation therapy, staphylococcal discitis, and Henoch-Schonlein purpur [22-24].

In our study, one patient with a rare case of congenital nephrotic syndrome had chylothorax; the mechanism was that chylous ascites in the peritoneum transferred to the pleural cavity through diaphragmatic defects because of the negative intrathoracic pressure during inspiration.

### 4.5. Treatment

The goal of the management of chylothorax is to relieve respiratory symptoms by drainage of the pleural fluid. After thoracocentesis is initially performed for diagnostic purposes, if the effusion is large and is compromising respiration, or there is a high possibility of recurrence, then a chest tube should be inserted for continuous drainage of the pleural space. The two most common agents aimed to reduce the production of chyle in patients with chylothorax are somatostatin and octreotide. Other agents include nitric oxide, etilefrine, tetracycline, talc, bleomycin, fibrin glue, and povidon-iodine, and OK-432 [4].

Surgical intervention, include thoracic duct ligation, pleurodesis, pleuroperitoneal shounts, pleurectomy, and pleural abrasion, sometimes become necessary when medical therapy fails. There is currently no consensus on the timing of surgery [2, 4]. However, most recommend a period 3 to 4 weeks of conservative management before undertaking surgical intervention [1, 2, 8].

All of the patients in our study were treated with conservative management initially, and chest tube drainages were required in 20 of them. Only 3 patients needed surgical intervention subsequently.

### 4.6. Prognosis

The mortality rate from chylothorax has improved because of the more aggressive management plans that have been adopted [5]. The mortality rate is almost 10% in children with chylothorax developing after cardiac surgery, and 18% for those with neonatal diagnoses [9]. Malignant chylothorax, chronic debilitating chylothorax, and bilateral chylothoraces have unfavorable prognosises [25]. In our study, the mortality rate of patients with chylothorax was 18.2%; however, these patients died of underlying diseases that were unrelated to chylothorax.

## 5. Conclusion

In our study, the majority of chylothorax in children resulted from complications of cardiothoracic surgery, followed by congenital/ idiopathic, malignancy, and other less common conditions. Dignostic thoracocentesis for pleural fluid analysis should be done to provide clues for how best to manage chylothorax. The prognosis of chylothorax is good except for those cases where the cause is from other malignancies, which will lead to a high mortality rate.

## Conflicts of interest statement

The authors declare that there are no conflicts of interest.
